# *Trans*-Translation in *Helicobacter pylori*: Essentiality of Ribosome Rescue and Requirement of Protein Tagging for Stress Resistance and Competence

**DOI:** 10.1371/journal.pone.0003810

**Published:** 2008-11-26

**Authors:** Marie Thibonnier, Jean-Michel Thiberge, Hilde De Reuse

**Affiliations:** Institut Pasteur, Unité Postulante de Pathogenèse de Helicobacter, Paris, France; Centre for DNA Fingerprinting and Diagnostics, India

## Abstract

**Background:**

The ubiquitous bacterial *trans*-translation is one of the most studied quality control mechanisms. *Trans*-translation requires two specific factors, a small RNA SsrA (*tm*RNA) and a protein co-factor SmpB, to promote the release of ribosomes stalled on defective mRNAs and to add a specific tag sequence to aberrant polypeptides to direct them to degradation pathways. *Helicobacter pylori* is a pathogen persistently colonizing a hostile niche, the stomach of humans.

**Principal Findings:**

We investigated the role of *trans*-translation in this bacterium well fitted to resist stressful conditions and found that both *smpB* and *ssrA* were essential genes. Five mutant versions of *ssrA* were generated in *H. pylori* in order to investigate the function of *trans*-translation in this organism. Mutation of the resume codon that allows the switch of template of the ribosome required for its release was essential *in vivo*, however a mutant in which this codon was followed by stop codons interrupting the tag sequence was viable. Therefore one round of translation is sufficient to promote the rescue of stalled ribosomes. A mutant expressing a truncated SsrA tag was viable in *H. pylori*, but affected in competence and tolerance to both oxidative and antibiotic stresses. This demonstrates that control of protein degradation through *trans*-translation is by itself central in the management of stress conditions and of competence and supports a regulatory role of *trans*-translation-dependent protein tagging. In addition, the expression of *smpB* and *ssrA* was found to be induced upon acid exposure of *H. pylori*.

**Conclusions:**

We conclude to a central role of *trans*-translation in *H. pylori* both for ribosome rescue possibly due to more severe stalling and for protein degradation to recover from stress conditions frequently encountered in the gastric environment. Finally, the essential *trans*-translation machinery of *H. pylori* is an excellent specific target for the development of novel antibiotics.

## Introduction

*Helicobacter pylori* is a gram negative bacterial pathogen that infects the stomach of about half of the world population. *H. pylori* is mostly acquired during childhood and the infection persists during decades unless patients receive an eradication treatment. Persistent colonization is concomitant with a strong inflammation of the mucosal layer triggering gastric pathologies such as gastritis, duodenal and peptic ulcer, adenocarcinoma or MALT lymphoma [1]. Lifelong colonization of the gastric mucosa by *H. pylori* implies that this bacterium is well adapted to this hostile environment facing both permanent acid stress in the mucus layer and oxidative stress at the gastric epithelium due to the host's immune response [2]. The mechanisms involved in the recovery from damages caused by the exposure to stress are critical in the adaptive response. These involve both active repair procedures (well studied for oxidative stress in *H. pylori* [3]) and quality control mechanisms.

In the present study, we addressed the role of *trans*-translation in *H. pylori*. *Trans*-translation is one of the most studied quality control mechanisms that provides bacteria with a general surveillance of the flow of genetic information [4], [5], [6], [7]. This mechanism rescues ribosomes sequestered on defective mRNAs lacking appropriate termination signals hence unable to efficiently resume the translation process. In addition, *trans*-translation promotes decay of these defective mRNAs and adds an amino acid tag to the truncated proteins to direct them to degradation pathways. *Trans-*translation relies on the properties of SsrA, a small stable RNA also called *tm*RNA, which shares features with a tRNA and a mRNA [5], [6]. Studies mainly performed on the *E. coli* system established the following mechanism of *trans*-translation. First, alanylated SsrA forms a complex with essential protein partners SmpB and EF-Tu and acts as a tRNA by allowing the nascent polypeptide encoded by the defective mRNA to be transferred onto the tRNA^Ala^-like domain of SsrA. Then, a short coding sequence within SsrA, referred to as the tag sequence behaves like a surrogate mRNA. This tag sequence provides the stalled ribosome with a new template for translation that is terminated by an in-frame stop codon; thus, allowing the release of recyclable ribosomal subunits, and the addition of a C-terminal tag to the nascent peptide. These tagged *trans*-translated polypeptides are specifically targeted to mainly ATP-dependent proteases [8], [9] and the defective mRNAs are degraded by RNase R [10].

While the overall mechanistic of *trans*-translation and the origin of defective or broken mRNAs have been extensively studied, questions on the precise biological role of this system are only partially answered. It was shown that normally growing cells undergo frequent *trans*-translation events [11]. In addition, there appears to be some specificity in the proteins tagged by *tm*RNA under normal growth conditions. [12]. Situations favoring stalling of ribosomes which are shown to require *trans*-translation are typically use of miscoding antibiotics [13], premature transcription termination or ribonucleolytic cleavage by RNases. Although stress or starvation are thought to enhance the amount of defective mRNAs, little is known about the actual damages occurring to ribonucleic acids under these conditions. The general assumption is that these damages are similar to those of DNA molecule i.e. generation of base adducts upon alkylation [14] or single stranded-breaks due to ROS (Reactive Oxygen Species) [15].

Genes encoding SsrA and its protein co-factor SmpB are conserved among bacteria [4] and are generally dispensable. Surprisingly, despite this conservation no common physiological function of *trans*-translation was found in the different bacterial systems studied. Mutants defective in *trans*-translation exhibit a wide range of phenotypes related to regulation of cellular physiology, cell cycle timing, stress response or virulence [7], [16]. The precise reason why *trans*-translation is associated with these functions is rarely understood. In *E. coli*, inactivation of *ssrA* leads to reduced growth rate, delayed recovery from carbon starvation and temperature sensitivity [17], [18]. In *Bacillus subtilis*, *tm*RNA dependent growth was shown during temperature or chemical stress conditions that correlated with an increase of the cellular amounts of SsrA [19]. *E. coli, Salmonella* or *Synechocystis* strains defective in *trans*-translation were found to be hypersensitive to different antibiotics [20], [21]. Deficiency in *trans*-translation affects the ability of *Salmonella enterica* serovar Typhimurium to colonize mice [22] and to survive within macrophages [23]. A Δ*ssrA-smpB* mutant of *Yersinia pseudotuberculosis* is avirulent in a mouse infection model, this is due to a loss in the induction of known virulence factors (motility, Type 3 secretion system) [24]. *tm*RNA also has a regulatory role for the correct timing of cell cycle regulation of *C. crescentus* [16]. Interestingly, SsrA with a protease-resistant SsrA tag does not restore motility or proper DNA replication in *Y. pseudotuberculosis* and *C. crescentus*, respectively [24], [25]. However, the role of the tag in stress response was never investigated.

The essentiality of the *smpB* gene has been deduced from systematic gene interruption studies performed in only three organisms *Mycoplasma genitalium* [26], *Mycoplasma pulmonis* [27] and *Haemophilus influenzae* [28]. Essentiality of *ssrA* was only demonstrated in *Neisseria gonorrhoeae* [29]. Due to the difficulties in studying essential functions inside the cells, little is known about *trans*-translation in species in which it is required for *in vitro* growth. Only in *N. gonorrhoeae* was this phenotype analyzed further. It was demonstrated that the essential function of *trans*-translation is the ribosome rescue whereas tagging activity was dispensable [29].

We decided to investigate the role of *trans*-translation in *H. pylori* because this bacterium is permanently subjected to stressful conditions that could increase the occurrence of premature transcription termination events. While the predicted *H. pylori tm*RNA structure and essential residues were conserved in comparison with those of the well-studied molecule of *E. coli*, the tag sequence of *H. pylori* presented some striking differences. This manuscript presents the demonstration of the essential character of *trans*-translation during *in vitro* growth of *H. pylori* and the investigation of its functional characteristics by site directed mutagenesis. We showed that residues necessary for ribosome rescue by SsrA are essential for *H. pylori* growth and that the tagging of *trans*-translated proteins is required for its adaptation to stressful conditions and for competence.

## Results

### *smpB* and *ssrA* are essential genes in *H. pylori*

**Figure 1:**
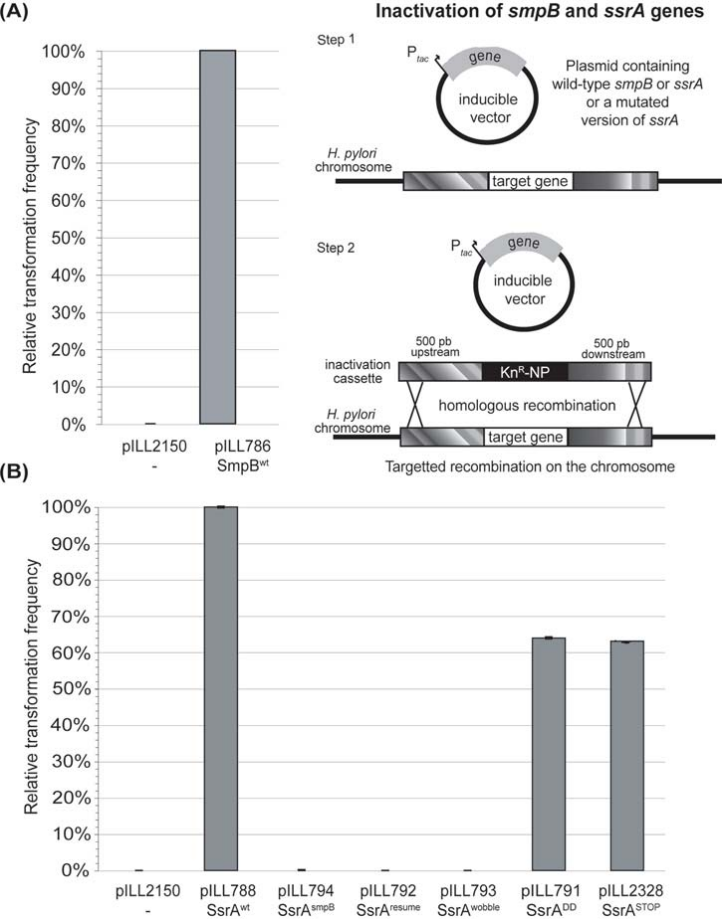
Inactivation strategy and measurements of relative transformation frequency of *H. pylori* strain N6 harboring different plasmids: A) pILL786 carrying wild-type *smpB*, or B) pILL788 carrying wild-type *ssrA* or different plasmids with mutagenized versions of *ssrA* (short names of the mutations are indicated) by suicide plasmids designed to create chromosomal deletions of *smpB* or *ssrA*, respectively. A strain carrying the empty vector pILL2150 served as a negative control. The transformation frequency is calculated as the number of transformants obtained for 5×10^8^ cells and 1 µg of DNA and expressed as a percentage of that by plasmids with wild-type *smpB* or *ssrA.*

Attempts to inactivate the *smpB* and *ssrA* genes encoded by *hp1444* and *hp0784*, respectively, in several *H. pylori* backgrounds were repeatedly unsuccessful suggesting that these genes and the *trans*-translation process are essential for *in vitro* growth of *H. pylori*. In parallel, gene *hp1248* predicted to encode RnaseR was deleted showing that this function is dispensable in *H. pylori*. To formally demonstrate the essentiality of *ssrA* and *smpB*, *H. pylori* strain N6 was first transformed by stably replicating plasmids pILL788 and pILL786 expressing *ssrA* or *smpB*, respectively, under control of an IPTG inducible promoter derived from pILL2150 [30] (Tables 1 and S1). Deletions of the *ssrA* chromosomal copy of strain N6 pILL788 and the *smpB* chromosomal copy of strain N6 pILL786 were obtained after transformation by suicide plasmids in the presence of IPTG as illustrated in Figure 1. These suicide plasmids carried a kanamycin resistance cassette flanked by DNA regions situated immediately upstream and downstream of the genes to be inactivated, thereby forcing homologous recombination outside the coding sequences of *ssrA* or *smpB* and thus specifically targeting allelic exchange into the chromosomal gene copy. When N6 carrying the empty vector pILL2150 was transformed with either of the two suicide plasmids, we obtained either no kanamycin resistant clones or a couple of clones that were either non viable or had undergone illegitimate recombination (Fig. 1). This demonstrated that *ssrA* and *smpB* are essential genes in *H. pylori* and prompted the investigation of their roles in this pathogen.

**Table 1:** Plasmids used in this study

Plasmid designation	Relevant genotype and properties	Source or reference
pILL2150	*H. pylori*/*E. coli* shuttle vector containing an inducible promoter	[30]
pILL796	Suicide vector containing the *ssrA* inactivation cassette	this work
pILL786	*smpB* cloned into pILL2150	this work
pILL788	wild-type *ssrA* cloned into pILL2150	this work
pILL791	*ssrA* ^DD^ derivative of pILL788	this work
pILL792	*ssrA* ^resume^ derivative of pILL788	this work
pILL793	*ssrA* ^wobble^ derivative of pILL788	this work
pILL794	*ssrA* ^smpB^ derivative of pILL788	this work
pILL2328	*ssrA* ^STOP^ derivative of pILL788	this work
pILL2322	*hypB-tap* fusion with stop codon cloned into pILL2150	this work
pILL2323	*hypB-tap* fusion without stop codon cloned into pILL2150	this work
pILL2332	*amiF* terminator introduced into pILL2322	this work
pILL2333	*amiF* terminator introduced into pILL2323	this work

### SmpB depletion in *H. pylori* results in growth arrest

**Figure 2:**
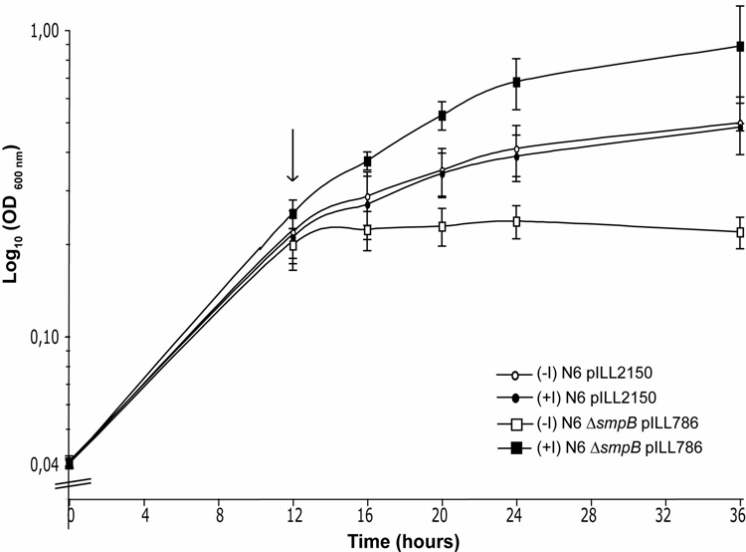
The effect of SmpB depletion on the growth kinetics of *H. pylori* strains. The following strains N6 pILL2150 (empty vector), N6Δ*smpB* pILL786 (vector expressing the SmpB protein under control of an inducible promoter P_tac_) were grown with the inducer IPTG 1 mM (+I) or without inducer (-I). An arrow indicates the arrest of growth of strain N6Δ*smpB* pILL786. The standard deviations for 5 different measurements are shown by error bars.

Construction of strain N6Δ*smpB* pILL786 and strain N6Δ*ssrA* pILL788 provided us with valuable *H. pylori smpB* or *ssrA* conditional mutants. The impacts of SmpB or SsrA depletion on *H. pylori* growth was measured. Notably, SsrA stability has been shown to be diminished in the absence of SmpB [31]. After approx. two doubling times in liquid medium without inducer, the conditional *smpB* mutant stopped dividing as a consequence of SmpB depletion (Fig. 2). Interestingly, we observed that SmpB depletion stops bacterial division but does not cause cell death growth. Indeed, the SmpB conditional mutant could be rescued if plated in the presence of the inducer IPTG (until 24 h growth, data not shown). In contrast, after 24 h, growth rescue was not possible suggesting that SmpB-depleted bacteria had undergone a physiological switch irreversibly directing them towards bacterial death.

**Figure 3:**
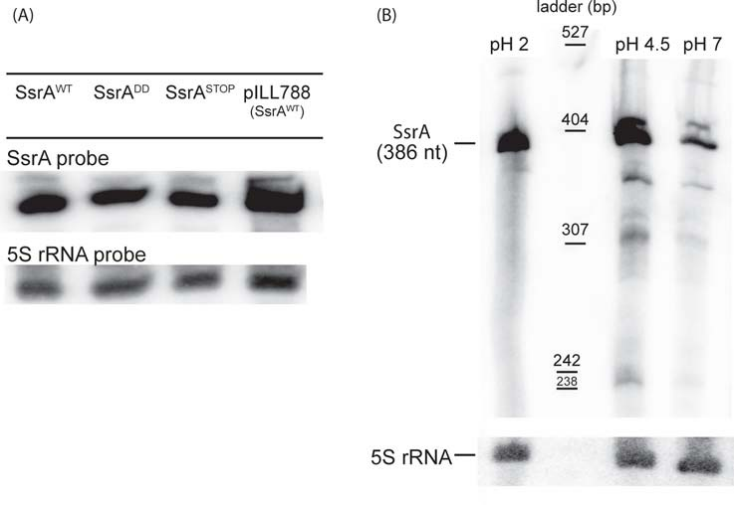
Northern blot analysis of total RNA extracted from *H. pylori* 26695 strain or different isogenic mutants using a *ssrA* riboprobe. Panel A: *H. pylori* expressing wild type SsrA, different mutant versions of SsrA or over-expressing wild type SsrA from plasmid pILL788. Panel B: *H. pylori* wild type strain incubated at different pH values. Normalization was performed with 5S rRNA probes. The ladder corresponds to DNA of pBR322 plasmid digested by *Msp*I, labeled and denaturated.

Similar experiments with the SsrA conditional mutant did not result in observable bacterial growth arrest. We concluded that the number of bacterial divisions occurring between the inoculation and the entry of the cells into stationary growth phase was not sufficient to dilute the intracellular concentration of SsrA to a level critical for cell growth. Indeed, northern blots revealed significant SsrA over-expression in *H. pylori* strain N6Δ*ssrA* pILL788 (Fig. 3A). In agreement with this interpretation is the documented high stability of SsrA molecules [18].

Upon IPTG induction, both SmpB and SsrA conditional mutants presented a slightly higher growth rate when compared with the wild type strain (for SmpB, see Fig. 2). This suggested that enhancing the *trans*-translation process improves the fitness of *H. pylori* under these conditions.

### Site directed mutagenesis of *H. pylori ssrA*

**Figure 4:**
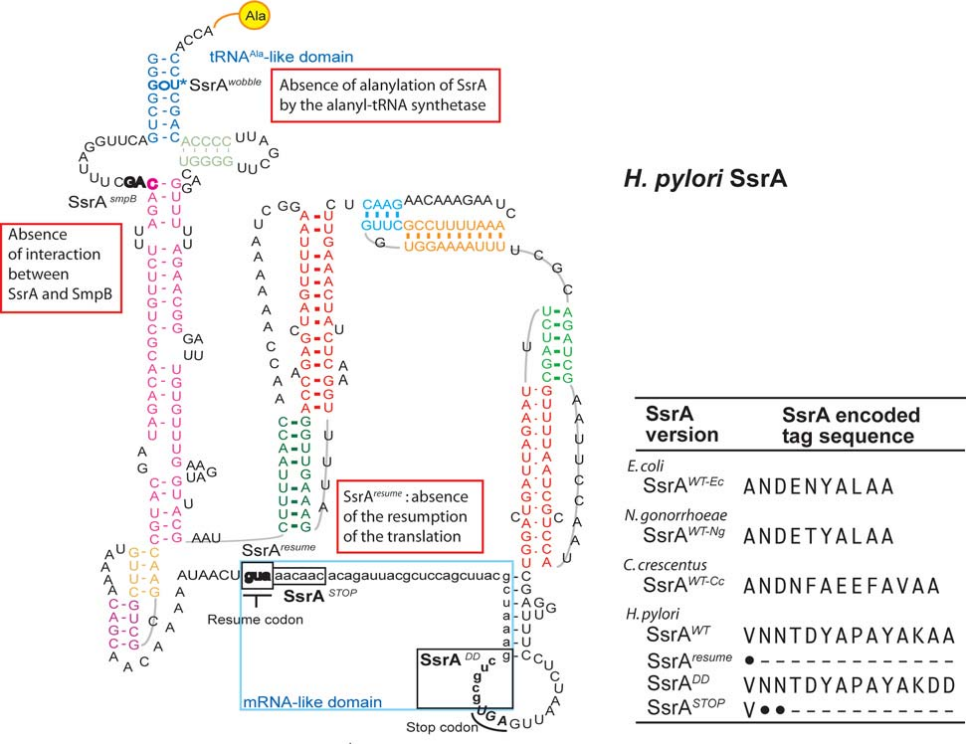
Putative model of the Helicobacter pylori mature tmRNA after the tmRNA web site (http://www.indiana.edu/~tmrna/). The positions of the mutations studied in this work are emphasized in the figure. Designation of the mutations and targeted functions: SsrA^SmpB^: mutation in the interaction site between SsrA and SmpB, G19U; A20U; C21A. SsrA^wobble^: mutation of the wobble G•U in the tRNA^Ala^-like domain, U380C. SsrA^resume^: substitution of the resume codon by a stop codon, G84U; U85A ; A86A. SsrA^DD^: substitution of the two terminal alanine codons of the tag by asparate codons. SsrA^STOP^: introduction of two stop codons downstream from the resume codon; A87U; C89A; A90U; C91G; C92A. A table is presented below that summarizes the encoded tag sequences of some bacteria and that indicates the product of the mutated SsrA tag sequences. Filled circles represent stop codons.

To investigate the different functions of SsrA in the *trans*-translation process, five different mutations were introduced in the *ssrA* gene on plasmid pILL788. Figure 4 illustrates a model of the *H. pylori* SsrA (*tm*RNA) molecule based on the predictions of the *tm*RNA web site (http://www.indiana.edu/~tmrna/). The residues for which a defined function in ribosome rescue was assigned in the well-studied *E. coli tm*RNA presented strong conservation (Fig. 4). Interestingly, the tag sequence from *H. pylori* showed several differences when compared with that of the previously studied *tm*RNAs from *E. coli*, *N. gonorrhoeae* or *C. crescentus*. The positions of the mutations analyzed in the present study have been emphasized in Fig. 4. The first two mutations targeted residues that were identified to be required for the interaction of SsrA with factors involved in the *trans*-translation process in *E. coli*. First, the predicted SmpB interaction site of SsrA [32] was modified by the introduction of three consecutive mutations G19U-A20U-C21A, and this mutant was designated SsrA^SmpB^. Second, the G•U mismatch in the tRNA^Ala^-like domain of SsrA was targeted. Recognition of this mismatch by the alanyl-tRNA synthetase is mandatory for the addition of Ala at the 3′ end of SsrA [33]. This mutant designated SsrA^wobble^ carried a U380C modification. Next, by substituting the resume codon GUA (positions 84-85-86) by a stop codon (UAA) the restart of translation was abolished. This type of mutant designated SsrA^resume^ was not tested before *in vivo* for essentiality in another organism. To specifically study the role of the *tm*RNA-dependent protein tagging in *H. pylori*, two different mutations in the tag region of *ssrA* were introduced. These mutations would uncouple the two functions of *tm*RNA, ribosome rescue and protein tagging for degradation. In one mutant, the two terminal codons of the tag region coding for Alanine were changed into Aspartate codons (SsrA^DD^ in Fig. 4). In *E. coli*, non polar residues in the C-terminus part of the tag (ALAA) are critical for recognition by cellular proteases [8], [9] and their mutation causes stabilization of the *trans*-translated peptides. We also wanted to examine the behavior of the *H. pylori* essential *tm*RNA under conditions in which a minimal tag was appended to the truncated peptides generated by *trans*-translation events. Therefore, the second and the third codons of the tag sequence were replaced by two stop codons (UAA-UGA), the mutant was designated SsrA^STOP^.

### Identification of essential residues in the *H. pylori tm*RNA

Plasmids carrying mutated *ssrA* were introduced into *H. pylori* strain N6 (Table 1). To evaluate the impact of these mutations on *H. pylori* viability, these strains were transformed with the suicide plasmid pILL796 designed to delete the chromosomal copy of *ssrA* and the number of transformants on selective medium were counted (Fig. 1). The frequency of transformation was determined by calculating the number of transformants for a given amount of viable cells (5×10^8^ bacteria) with 1 µg DNA of the suicide plasmid (pILL796). The transformation frequency of the chromosomal deletion of *ssrA* in a recipient strain N6 carrying the wild type *ssrA* plasmid (pILL788) was estimated to be 2×10^−3^. Identical transformation frequencies of strains carrying pILL791 with SsrA^DD^ and pILL2328 with SsrA^STOP^ were obtained that were similar to that of N6 with SsrA^wt^ (Fig. 1 B). The frequency of transformation was at least four orders of magnitude lower for the inactivation attempts of strains carrying one of the three mutations affecting the ribosome rescue process, SsrA^resume^ (pILL792), SsrA^wobble^ (pILL793) and SsrA^smpB^ (pILL794) (Fig. 1 B). This data showed that each of these essential steps of the *trans*-translation process is essential in *H. pylori*. In contrast, the mutations affecting the tag do not impact bacterial viability. Importantly, viability of the SsrA^STOP^ mutant appending a minimal tag (Ala from *tm*RNA and Val from the resume codon) suggests that one round of translation is sufficient to rescue the stalled ribosomes. This latter mutant allowed us to evaluate the role of protein tagging *in vivo* under conditions that were more drastic than the point mutations affecting tag recognition described in previous studies.

### Mutations in the tag of the *tm*RNA are viable in *H. pylori* and do not affect *in vivo* colonization

To analyze the phenotype of *H. pylori* mutants with a modified *tm*RNA tag, SsrA^DD^ and SsrA^STOP^ mutations were introduced by allelic exchange into the chromosome replacing the wild type *ssrA* alleles in three different *H. pylori* backgrounds N6, X47-2AL and 26695 (Table S1). N6 is a strain in which the shuttle plasmid replicates in a stable manner, X47-2AL is a mouse-adapted strain and 26695 is a strain from which the entire genome has been sequenced. The expression level and stability of the mutated versions of SsrA in *H. pylori* strain 26695 were identical to that of the wild type SsrA (Fig. 3A).

Mutants were obtained in every strain as expected (Table S1) and their growth under normal conditions was not affected. These strains were used to evaluate the role of tagging under several conditions relevant to the gastric niche of *H. pylori* such as growth at pH 5.5 (mutants of strain 26695), motility and colonization of a mouse model (mutants of strain X47-2AL) (data not shown). The mutants behaved like the corresponding isogenic wild type strains under the conditions tested. It was concluded that the tagging process of *trans*-translation is not essential for *in vivo* survival and motility of *H. pylori*.

### Assessment of SsrA mediated protein tagging in *H. pylori* strains expressing mutant SsrA versions

**Figure 5:**
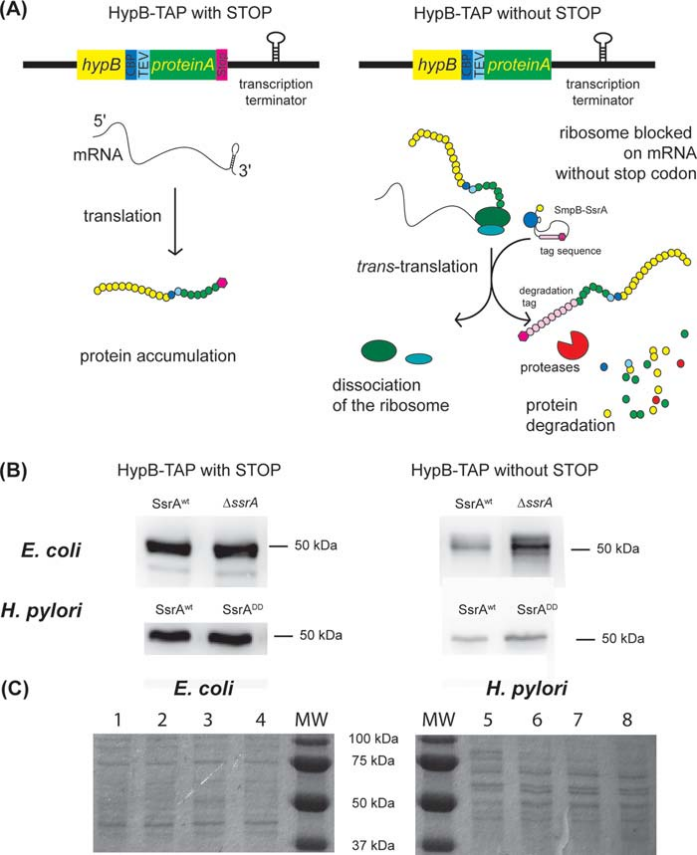
Use of an artificial *trans*-translation target to measure protein degradation in wild-type strains and SsrA mutants. A) Construction strategy of the reporter genes and predicted fate of the encoded proteins. B) Western-blots of whole cell extracts from *E. coli* or *H. pylori* strains expressing HypB-TAP with and without the terminal stop codon (MM 47 kDa) and revealed by peroxidase coupled anti-peroxidase antibody that binds to the protein A motif. C) Loading controls are presented: line 1 and 5 correspond to crude extracts of SsrA wild type *E. coli* and *H. pylori* strains expressing HypB-TAP with STOP respectively, line 2 and 6 correspond to whole extracts of Δ*ssrA E. coli* and SsrA^DD^ *H. pylori* mutants expressing HypB-TAP with STOP respectively, line 3 and 7 correspond to the whole extracts of SsrA wild type *E. coli* and *H. pylori* strains expressing HypB-TAP without STOP respectively and line 4 and 8 correspond to whole extracts of Δ*ssrA* WT *E. coli* and SsrA^DD^ *H. pylori* mutants expressing HypB-TAP without STOP respectively.

To examine the actual protein tagging activities in *H. pylori*, we engineered a pair of artificial *trans*-translation target proteins (Fig. 5 A) composed of a fusion between the non-essential gene *hypB* (coding for *H. pylori* hydrogenase accessory protein) and a sequence encoding protein A from *Staphylococcus aureus* that could easily be detected by western blotting. This gene fusion designated *hypB-TAP* is described in Stingl *et al.* [34]. Our aim was to evaluate the fate of these target proteins when expressed in *H. pylori* mutants defective in tagging activity. Therefore, two constructions were generated, one was terminated by a translational stop codon and the other devoid of a stop codon, both were followed by a transcriptional terminator. Western blots in *E. coli* (Fig. 5 B) indicated that these constructs behaved like efficient *trans*-translation tagging target proteins. In *E. coli* MG1655 wild-type strain, the protein fusion with stop (expressed by pILL2332) was expressed in large amounts while that without stop (pILL2333) was less present indicating that protein degradation had occurred (Fig. 5 B). Involvement of *trans*-translation in the degradation of HypB-TAP fusion without stop was demonstrated by the strong stabilization of this protein in an *E. coli* Δ*ssrA* strain (Fig. 5 B) in contrast to the amounts of the fusion with stop that were unchanged. Given the large Molec Mass (50 kDa) of the HypB-TAP fusion, addition of the 1.5 kDa tag by *trans*-translation was not visible on an acrylamide gel.

We then decided to introduce by natural transformation the two reporter genes *hypB-TAP* with or without stop either expressed from plasmids (pILL2332 and pILL2333) or directly by recombination on the chromosome of *H. pylori* N6 wild type strain and of each of the two tag mutants. The wild type strain was transformed by the constructs at expected frequencies. In contrast, transformation efficacy was repeatedly diminished in both SsrA mutants; SsrA^DD^ strain presented a three fold lower efficacy and no transformants were obtained in the SsrA^STOP^ background (three independent experiments). Similar observations were made when a suicide plasmid targeting allelic exchange into *ureA-B* (described in [35]) was used as a control. The loss of competence of the SsrA^STOP^ mutant was unexpected and suggested that *trans*-translation dependent tagging is required for natural transformation in *H. pylori*.

As in *E. coli*, we found that in *H. pylori* wild type strain, HypB-TAP without stop expressed from the chromosome was heavily degraded (Fig. 5B) as compared to HypB-TAP with stop suggesting that it was indeed targeted by *trans*-translation. The SsrA^DD^ mutant only marginally stabilized the HypB-TAP without stop protein (about two fold) indicating that it was still subject to proteolysis.

### Minimal *trans*-translation-dependent protein tagging leads to increased sensitivity of *H. pylori* to antibiotics and oxidative stress

**Figure 6:**
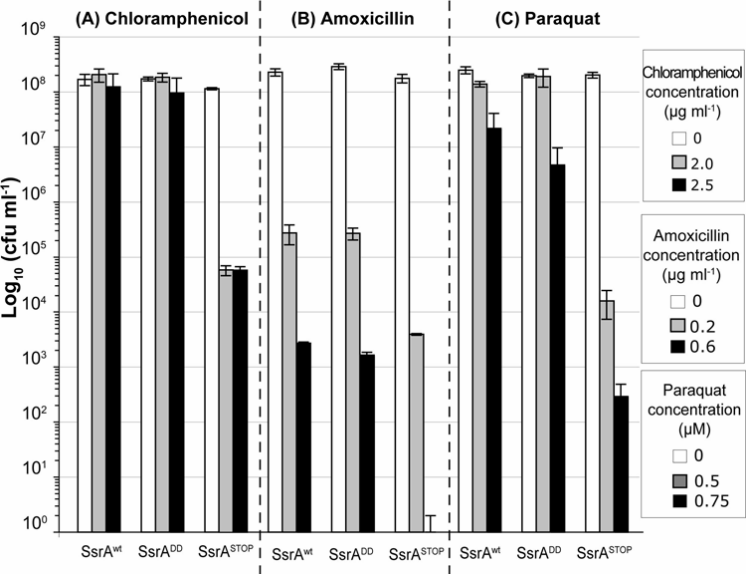
Increased susceptibility to sub-lethal doses of antibiotics, chloramphenicol (A) and amoxicillin (B) and high sensitivity to oxidative stress generated by paraquat (C) of *H. pylori* SsrA mutants defective in *trans*-translation tagging.

The role of the *trans*-translation dependent protein tagging in *H. pylori* strain 26695 after exposure to two types of stresses was addressed (Fig. 6). First, susceptibility to sub-lethal doses of two antibiotics was examined (*i*) chloramphenicol, a peptidyl transferase inhibitor that targets the translation machinery and, (*ii*) amoxicillin that irreversibly binds to the active site of penicillin-binding proteins (PBPs) involved in cell wall biosynthesis. Amoxicillin is one of the recommended components of the triple therapy employed in anti-*H. pylori* treatment. Second, the response of the mutants to oxidative stress was tested by measuring the sensitivity to paraquat (methyl viologen) that generates superoxide radicals. Superoxide radicals are among the molecules synthesized during the oxidative burst of immune cells. The SsrA^DD^ mutant behaved like the wild type strain during exposure to chloramphenicol, amoxicillin and paraquat (Fig. 6). In striking contrast, SsrA^STOP^ presented an enhanced sensitivity to chloramphenicol stress for doses of 2.0 and 2.5 µg.ml^−1^ and to amoxicillin with lethality at 0.6 µg.ml^−1^ (Fig. 6). In addition, the SsrA^STOP^ mutant that has a minimal tag sequence presented higher sensitivity to oxidative stress upon exposure to paraquat (Fig. 6).

### Acid stress causes induction of both *ssrA* and *smpB*

The association of *trans*-translation with the response to stress and the continual exposure of *H. pylori* to the acidity of its gastric niche lead us to ask whether this mechanism could provide the cell with a rapid adaptive response to stressful conditions. In a previous transcriptomic study, we detected *smpB* gene induction upon acid exposure of *H. pylori* strain 26695 [36]. Acid activation of *smpB* was validated with RT-PCR [36] and more recently by Northern blotting analysis (data not shown). *ssrA* messenger RNA was examined by Northern blots on total RNA extracted from exponential growing *H. pylori* cells (strain 26695) incubated for 30 min at pH 7, pH 4.5 or pH 2. We observed a band that corresponded to a molecule of 386 nt which is the length expected for a mature SsrA (as predicted from the 26695 genome sequence) (Fig. 3B). For bacteria exposed to pH 2 and pH 4.5, this band was significantly more intense than for bacteria exposed to pH 7 (Fig. 3B). This suggests that in *H. pylori*, SsrA amounts are increased at low pH.

## Discussion

While the mechanistic and structural aspects of *trans*-translation and of *tm*RNA have been extensively studied, several questions remain concerning the biological role of this system. It was established that under normal growth conditions, a specific pattern of proteins are targeted by *tm*RNA [12]. Yet, the function of this process in the cell is not clear. The role of *trans*-translation in ribosome rescue under stress conditions has been demonstrated, although the importance of tagging truncated proteins was not known. In addition, the essentiality of *trans*-translation in some bacterial species is not understood. The two latter issues and the role of this quality control mechanism in the pathogen *H. pylori* was addressed due to its exceptional ability to persist in a harsh environment.

### Essentiality of *trans*-translation in *H. pylori* and in other organisms

Both *ssrA* and *smpB* were demonstrated to be essential in *H. pylori*. Using a conditional expression system, SmpB depletion in *H. pylori* cells resulted in growth arrest that was not associated with immediate cell death, that only occurred after 24 h depletion. This suggested that no irreversible process or toxic product accumulation occurred when *trans*-translation was inactivated. The reason why *trans*-translation is essential in some organisms is still not understood but several hypothesis were raised. Essentiality of *trans*-translation has been proposed to be associated with small genomes or with the necessity to accurately manage a restricted pool of ribosomes expressed by a limited number of rRNA operons [29]. Table 2 summarizes the available data on *trans*-translation essentiality or dispensability in several bacteria with their genome size, the number of rRNA operons and the duplication time. It can be concluded that there is no correlation between any of these criteria and *trans*-translation essentiality. In particular, the proposed correlation between *trans*-translation essentiality and a reduced number of rRNA operons [29] has not been confirmed by this analysis. Slow growth rates that are associated with a reduced number of rRNA can also be excluded as a cause of *trans*-translation essentiality (Table 2). While essentiality was originally thought to be associated with small genomes, this notion is contradicted by the recent example of *Shigella flexneri* [7] rendering unlikely the hypothesis of the absence of an alternative mechanism for mRNA quality control in bacteria with reduced coding capacity. Other interpretations of *trans*-translation essentiality during normal growth conditions can be proposed. The accumulation of truncated proteins or mRNAs may be lethal or *tm*RNA-dependent tagging of a specific protein could be essential for bacterial survival. This was shown not to be the case in *H. pylori* since (i) tagging is not the essential function of *trans*-translation in *H. pylori* and, (ii) RnaseR, a conserved ribonuclease likely to be responsible for the degradation of defective messengers is dispensable.

**Table 2:** *Trans*-translation essentiality or dispensability in bacteria grown under normal conditions.

Organism	Size of the genome in Mb	Number of rRNA operons per genome	Duplication time in hours (Fast/Slow)	Status of *trans*-translation	Reference
*Helicobacter pylori*	1.65	2	2.4 (F)	Essential	This work
*Mycoplasma genitalium*	0.58	1	6 (S)	Essential	[26]
*Mycoplasma pulmonis*	058	1	2 (F)	Essential	[27]
*Neisseria gonorrhoeae*	1.8	4	1.1 (F)	Essential	[29]
*Haemophilus influenzae*	2.2	6	0.5 (F)	Essential	[28]
*Shigella flexneri*	4.6	7	0.35 (F)	Essential	Cited as non published data in [7]
*Escherichia coli* K12	4.6	7	0.35 (F)	Non-essential	[50]
*Salmonella enterica* serovar Typhimurium	4.8	7	0.4 (F)	Non-essential	SmpB [23] ssrA [22]
*Bradyrhizobium japonicum*	8.7	1	20 (S)	Non-essential	[51]
*Caulobacter crescentus*	4.0	2	1.5 (F)	Non-essential	[52]
*Streptomyces lividans*	8.6	6	4.2 (S)	Non-essential	[53]
*Yersinia pseudotuberculosis*	4.8	7	1.25 (F)	Non-essential	[24]
*Bacillus subtilis*	4.2	10	0.43 (F)	Non-essential	[19]

The genome size, number of copies of rRNA operons, duplication time (information kindly provided by E. Rocha) and relevant references are also indicated.

Interestingly, we observed that over-expression of either SsrA or SmpB enhances the *in vitro* growth rate of *H. pylori* suggesting an increase in the fitness of the bacterium under these normal conditions. In *B. subtilis*, while *trans*-translation is not essential under normal growth conditions, cells grew depending on the expression level of SsrA under stress conditions such as high temperature [19]. Therefore, it can be proposed that (i) *H. pylori* cells grown *in vitro* are submitted to some type of stress that produces damaged RNAs at a high occurrence and/or causes frequent ribosome pausing and, (ii) that in this bacterium, *trans*-translation components represent a limiting factor for normal growth. This could be related to the fact that *H. pylori* has intrinsically an elevated mutation rate compared to most other bacteria [37].

### Ribosome rescue with an intact resume codon is an essential function of *trans*-translation in *H. pylori*

The essentiality of several point mutations in *ssrA* was tested in *H. pylori*. Mutations in the SsrA tag sequence of *H. pylori* were viable. The lethality of SsrA mutations affecting the tRNA^Ala^-like domain (wobble), the interaction with SmpB and the resume codon for the restart of translation after ribosome stalling indicated that, in *H. pylori*, rescue of stalled ribosomes by *trans*-translation is essential. The two latter mutations were particularly interesting, since they were never tested *in vivo* for essentiality. *In vitro* studies showed that resume of the translation is mandatory for the dissociation of the stalled ribosome [38]. However, here we show that a single ribosomal translocation step is sufficient to allow its recycling since the mutant carrying stop codons instead of the second and third codons of the tag (SsrA^STOP^) is viable. In *N. gonorrhoeae*, the essential function of *trans*-translation was also associated with ribosome rescue and not with protein tagging [29].

### Viability of *H. pylori* mutants with a minimal *tm*RNA tag sequence

While mutation of the resume codon was lethal in *H. pylori*, introduction of two stop codons immediately after this position that restricted the added tag to only two amino acids did not affect *H. pylori* growth under normal conditions. This provided us with a valuable tool to examine *in vivo* the role of tagging of truncated peptides generated under conditions of functional *trans*-translation. Mutations in the tag sequence are expected to stabilize these peptides by preventing their recognition by specific proteases well defined in *E. coli* [8] and *C. crescentus* [39] and conserved in *H. pylori* [40]. The two last Ala codons of the tag (Fig. 4) have been reported to be critical for this recognition in several organisms. *H. pylori* SsrA^DD^ strain carrying such a mutation only weakly stabilized the artificial *trans*-translation target protein (HypB-TAP). We concluded that in contrast to what was described in *E. coli* or *B. subtilis,* these two conserved codons of the tag are not central for protease recognition in *H. pylori*. In addition, the *H. pylori* tag sequence presents two striking differences with those of *E. coli*, *B. subtilis*, *N. gonorrhoeae* and *C. crescentus* that could reflect differences in the degradation process. This includes the presence of a polar residue at the ante penultimate position in the last four amino acids of the proteolysis tag (A**K**AA in *H. pylori* instead of A**L/V**AA, Fig. 4) and the absence of a SspB recognition motif, a proteolytic adaptor predicted to be absent in *H. pylori*.

### Role of the *trans*-translation dependent tagging under stress conditions and for efficient DNA transformation

An original outcome of this study came from our observation that under conditions of functional ribosome rescue, the tagging of *trans*-translated protein was necessary for stress resistance and competence. Till now, in other organisms only mutants carrying deletions of the entire *tm*RNA or of *smpB* (deficient in both *trans*-translation functions) were examined for stress sensitivity.

The *H. pylori* SsrA^STOP^ mutant presented a multifaceted phenotype including (i) increased susceptibility to sub-lethal doses of chloramphenicol, (ii) hypersensitivity to amoxicillin, and (iii) deficient natural transformation capacity. In agreement with our previous conclusions, these phenotypes were not or only very marginally displayed by the SsrA^DD^ mutant.

In *E. coli,* sub-lethal concentrations of miscoding antibiotics such as kanamycin are known to enhance SsrA protein tagging activity due to translational read-through at normal stop codons, however read-through rarely occurs with chloramphenicol [13]. Alternatively, translation velocity reduction by chloramphenicol might increase the amount of cleaved mRNAs and thus the recruitment of *tm*RNA [5]. Bactericidal antibiotics such as amoxicillin targeting the cell wall synthesis are obviously not directly interfering with translation. Increased sensitivity to ampicillin for an *E. coli ΔssrA* mutant has been reported [21] while mutants of *Synechocystis* or *Y. pseudotuberculosis* did not display this phenotype [24], [41]. This class of antibiotics have recently been shown to stimulate production of hydroxyl radicals that damage nucleic acids including mRNA and therefore might indirectly require *trans*-translation [42]. The continual oxidative stress encountered by *H. pylori* at its colonization site represents a major challenge despite *H. pylori* being well-equipped to protect itself from ROS [43]. The SsrA^STOP^ mutant exhibits a striking hypersensitivity to ROS. Importantly, these results demonstrate for the first time that the tagging process is by itself important for the response to stress conditions. Stress could enhance the amount of truncated mRNAs either directly or through ribosome pausing and, as a consequence produce toxic accumulation of truncated untagged peptides. Alternatively, recovery from stress conditions might require *trans*-translation of specific proteins.

We found a novel role of *trans*-translation in natural transformation competence that, in *H. pylori*, depends on the *comB* Type IV secretion system [44]. Our results point to the need of *trans*-translation tagging of a specific protein required for efficient activity of this system. Noteworthy, *trans*-translation deletion mutants of *Y. pseudotuberculosis* are deficient in the delivery of Yop proteins by a Type III secretion system [24]. The existence of a common *trans*-translation dependent check-point mechanism required for the assembly of these two secretion systems is an attractive hypothesis that will need further investigation.

### SsrA in *H. pylori*: a one piece molecule induced during acid stress

Using RACE (Rapid Amplification of cDNA Ends) mapping for SsrA of *H. pylori*, Dong *et al.* [45] obtained two bands that were interpreted as indicative of a two-piece *tm*RNA like in *C. crescentus* and all the related α-proteobacteria [45], [46]. In contrast, northern blotting experiments presented in this study indicated that SsrA is an abundant one-piece molecule in *H. pylori*. In cells grown at neutral pH, SsrA was detected as one band with molecular weight corresponding to the size of the mature form (386 nt). A significant increase in the amounts of SsrA was observed in *H. pylori* cells exposed at low pH similar to that encountered in the gastric environment. Interestingly, expression of *smpB* is also induced by acidity [36]. Thus, in *H. pylori* the expression of the two effectors of *trans*-translation is higher upon acid exposure suggesting that this mechanism is enhanced under this stress condition.

We conclude that *trans*-translation is critical in *H. pylori* probably because this system is frequently required for ribosome rescue and that the associated protein tagging plays a regulatory role in the bacterial response to adverse conditions.

Finally, as more and more *H. pylori* strains present resistances to the commonly used antibiotics against this pathogen, we propose the essential *trans*-translation as an alternative specific target for the development of antibacterial drugs.

## Materials and Methods

### Bacterial strains and growth conditions

*Escherichia coli* strain MC1061 was used as a host for the preparation of plasmids employed to transform *H. pylori*. Antibiotics for the selection of recombinant *E. coli* strains were kanamycin (20 µg ml^−1^) or chloramphenicol (30 µg ml^−1^). The *H. pylori* strains were X47-2AL, 26695 and N6 (Table S1). *H. pylori* were grown as previously described [47]. Liquid cultures were grown in Brain Heart Infusion (BHI) (Oxoid) or Brucella broth (Difco) supplemented with 0.2% β-cyclodextrin and an antibiotics/fungicide mix. For growth kinetics, we inoculated a liquid pre-culture in Brucella Broth with 1 mM IPTG (isopropyl-β-D-thiogalactoside) using 24 h old plate-grown *H. pylori* strains at an initial OD_600nm_ of 0.15. Eight hours later, this preculture was diluted to a final OD 0.05, split into two flaks, one with IPTG 1 mM and one without and the cultures were followed during 35 H.

### Molecular techniques

Standard procedures were as in [48]. The QiaAmp DNA extraction kit (Qiagen) was used to extract chromosomal DNA from *H. pylori*. Amplifications for mutagenesis were performed using the Long Template PCR system (Roche). Oligonucleotides used for PCR amplification, site directed mutagenesis or sequencing are listed in Table S2.

### Construction of *H. pylori* plasmids and mutants

Plasmids are listed in Table 1, strains in Table S1. pILL2150 is a *H. pylori*/*E. coli* shuttle vector carrying an inducible P_tac_ promoter that is functional in *H. pylori* [30]. pILL786 and pILL788 were obtained by cloning wild-type *smpB* and *ssrA* genes in pILL2150, respectively. Transformation by pILL2150 and its derivates was selected on 8 µg ml^−1^ chloramphenicol. Deletions and point mutations were introduced in *H. pylori* strains by allelic exchange using suicide plasmids or three-step PCR products (as in [49]) in which *H. pylori* DNA regions corresponding to the gene to be mutated are flanking a non-polar kanamycin resistance cassette [35]. These plasmids or PCR products were introduced in *H. pylori* by natural transformation and selection for mutants that had undergone double crossing-over events was performed with 20 µg ml^−1^ kanamycin [47].

Inactivation of chromosomal genes in *H. pylori* were performed for *smpB* and *hp1248* (encoding RnaseR) with a three-step PCR product as in [49] and for *ssrA* with the suicide plasmid pILL796 as in [47]. Site-directed mutagenesis was performed on pILL788 as in [47]. Correct chromosomal insertion of a non-polar kanamycin cassette in *hp1248*, *smpB* (*hp1444*) and *ssrA* (*hp0784*) was verified by PCR and the introduction of point mutations in *ssrA* were checked by sequencing as in [47].

### Construction of the *hypB-TAP* reporter gene and western blotting

To assess the efficacy of *trans*-translation mediated protein degradation, a reporter HypB protein fused to a TAP-tag (Tandem Affinity Purification tag) was used [34]. HypB, a hydrogenase accessory protein encoded by *hp0900,* is fused with a C-terminal tag containing two tandem protein A regions that can be detected very specifically in *H. pylori* extracts by western blot. *hypB-tap* was PCR amplified from the original construction 26695-*hypB*-*TAP* [34] with either oligonucleotides H359/H340K that include the stop codon situated at the end of the tag sequence or with oligonucleotides H359/H341K that do not contain this stop codon. Both PCR products were cloned into pILL2150 [30] using the *Spe*I-*Kpn*I restriction sites generating plasmids pILL2322 (with stop) and pILL2323 (without stop). In a last step, using a *Kpn*I restriction site, we added the *amiF* (*hp1238*) transcriptional terminator downstream from the fusion genes using a *Kpn*I restriction site and obtained pILL2332 (fusion with stop and terminator) and pILL2333 (fusion without stop and with terminator), the constructions were verified by sequencing. Using three step PCR, these two fusions and the adjacent *cat* gene of the plasmid were amplified and introduced at the *hypB* chromosomal locus after transformation and chloramphenicol selection. Immunodetection of HypB-TAP proteins was performed on crude extracts migrated through SDS-PAGE with a peroxydase-coupled anti-peroxydase antibody (Sigma) as in [49]. Intensities were quantified with the Quantity One software (Bio-Rad).

### Measurement of transformation efficacy

*H. pylori* N6 strain harboring pILL786, pILL788, pILL791, pILL792, pILL793, pILL794, and pILL2328 (Table 1 and S1) were grown on blood agar plates, harvested after 24 h and suspended in peptone broth (Difco). Bacterial ODs were adjusted to OD 15, then 50–200 µl (approx 5×10^8^ cells) of these preparations were spotted in duplicates on non selective plates and left to grow. Four hours later, one patch was taken for enumeration in order to determine the number of viable bacteria. One µg of plasmid DNA or PCR product was added on the other patch in order to inactivate the chromosomal copies of *ssrA* or *smpB* genes, respectively. Twelve hours later these bacteria were plated on selective media, and four days later the total numbers of transformants were counted. Transformation rates represent the number of transformants obtained per viable cell for 1 µg of DNA.

### Motility tests and mouse model for colonization

*H. pylori* strain X47-2AL and its isogenic mutants expressing SsrA^DD^ and SsrA^STOP^ were grown on plates for 18 h and harvested in 500 µl of peptone broth (approx OD 15). To test the motility of the strain, 2 µl of the preparations were inoculated on Brucella Broth (Difco) soft–agar plates, 0.035% Bacto-Agar (Difco), 10% (v/v) decomplemented FCS (Eurobio) by piercing the agarose. The plates were left to grow for 7 days at 37°C. Motility was measured by determining the diameter of the spread around the inoculation spot.

The *in vivo* colonization capacities of *H. pylori* strain X47-2AL and its isogenic mutants expressing SsrA^DD^ and SsrA^STOP^ were assessed as in [36].

### Sensitivity tests

Overnight liquid cultures of wild type *H. pylori* strain 26695 or of the two isogenic tag- mutants SsrA^DD^ and SsrA^STOP^ were used to inoculate BHI medium containing 10 % FCS at an initial OD of 0.15 and left to grow for 6 h. Cultures growing exponentially were used to perform the following tests. Serial dilutions of the bacteria were spotted on plates containing different concentrations of chloramphenicol (Sigma) 2 or 2.5 µg ml^−1^; Amoxicillin (Clamoxyl, GlaxoSmithKline) 0.2 or 0.6 µg ml^−1^ and plates were incubated under microaerophilic conditions. The controls consist of culture grown without these antibiotics. Counting of surviving bacteria was performed 5 days later.

To determine the sensitivity of the strains to oxidative stress, 1 ml (approx 10^7^ bact) of cells in exponential phase were placed into 12-wells plates containing BHI supplemented with 0.5 or 0.75 µM paraquat (methyl Viologen, Sigma). Cultures were incubated at 37°C under microaerophilic conditions while shacking at 160 rpm. After 18 h, bacterial counts were performed on blood agar plates.

### RNA extraction and Northern blotting

Exponentially growing liquid cultures of *H. pylori* wild type 26695 or isogenic mutants (OD 0.6) were centrifuged at room temperature for 10 min at 3000 g. Pellets were suspended in preheated BHI medium adjusted to pH 2.0, pH 4.5 or pH 7.0 at an OD of 0.2, left for 30 min. RNA was extracted using the phenol-chloroform method [36]. Four µg of total RNA were separated on 4% acrylamide-urea denaturing gels, blotted onto Hybond-N+ membrane (Amersham) with a transblotter (40 min, 10 mV) and U.V. cross-linked. 5S rRNA and a 300-nucleotides-long internal fragment of *ssrA* ^32^P-labeled riboprobes were synthesized with the StripAble RNA Probe Synthesis and Removal Kit (Ambion). 5S rRNA probed on the same membranes served for calibration. Hybridization was performed at 65°C for 4 h with UltraHyb (Ambion). Quantitative analyses of blots were performed with Quantity One software (Bio-Rad).

## Supporting Information

Table S1 (0.10 MB DOC)

Table S2 (0.19 MB DOC)
