# Supplementary material for: Trans-Translation in Helicobacter pylori: Essentiality of Ribosome Rescue and Requirement of Protein Tagging for Stress Resistance and Competence
Source: PLoS One. 2008 Nov 26;3(11):e3810. doi: 10.1371/journal.pone.0003810 (PMC2584231; doi:10.1371/journal.pone.0003810)
Supplement: Table S1 — (0.10 MB DOC) [file pone.0003810.s001.doc]

Supplementary material

Table S1: Bacterial strains used in this study

| **Strain** | **Relevant genotype and properties** | **Source/Ref.** |
| --- | --- | --- |
| ***E.coli*** |  |  |
| MC1061 | Parental strain | [54] |
| MG1655 | Parental strain | [55] |
| MG1655 ∆*ssrA* | ∆*ssrA* | [18] |
| MG1655∆*ssrA* pILL2332 | *∆ssrA* mutant with pILL2332  carrying the *hypB-TAP* fusion with stop codon, Kn^R^ | this work |
| MG1655 ∆*ssrA* pILL2333 | *∆ssrA* mutant with pILL2333  carrying the *hypB-TAP* fusion without stop codon, Kn^R^ | this work |
| ***H. pylori*** |  |  |
| N6 | Parental strain | [56] |
| N6 pILL2150 | Parental strain carrying an *H. pylori*/*E. coli* shuttle vector, Cm^R^ | [30] |
| N6 pILL786 | N6 with pILL786 carrying *smpB*, Cm^R^ | this work |
| N6 pILL788 | N6 with pILL788 carrying wild type *ssrA*, Cm^R^ | this work |
| N6 pILL791 | N6 with pILL791 carrying *ssrA*^DD^, Cm^R^ | this work |
| N6 pILL792 | N6 with pILL792 carrying *ssrA*^resume^, Cm^R^ | this work |
| N6 pILL793 | N6 with pILL793 carrying *ssrA*^wobble^, Cm^R^ | this work |
| N6 pILL794 | N6 with pILL794 carrying *ssrA*^SmpB^, Cm^R^ | this work |
| N6 pILL2328 | N6 with pILL2328 carrying *ssrA*^STOP^, Cm^R^ | this work |
| N6 ∆*smpB* pILL786 | *∆hp1444* mutant with pILL786 carrying *smpB*, Kn^R^, Cm^R^ | this work |
| N6 ∆*ssrA* pILL788 | *∆hp0784* mutant with pILL788 carrying wild type *ssrA*, Kn^R^, Cm^R^ | this work |
| N6 *ssrA*^DD^ | Chromosomal mutation of the *ssrA* tag sequence DD, Kn^R^ | this work |
| N6 *ssrA*^STOP^ | Chromosomal mutation of the ssrA tag sequence STOP, Kn^R^ | this work |
| X47-2AL | Parental strain used for mouse colonization | [57] |
| X47-2AL *ssrA^DD^* | Chromosomal mutation of the *ssrA* tag sequence DD, Kn^R^ | this work |
| X47-2AL *ssrA^STOP^* | Chromosomal mutation of the *ssrA* tag sequence STOP, Kn^R^ | this work |
| 26695 | Sequenced parental strain | [40] |
| 26695 ∆*hp1248* | Chromosomal inactivation of *hp1248*, encoding Rnase R, Kn^R^ | this work |
| 26695 *ssrA^DD^* | Chromosomal mutation of the *ssrA* tag sequence DD, Kn^R^ | this work |
| 26695 *ssrA^STOP^* | Chromosomal mutation of the *ssrA* tag sequence STOP, Kn^R^ | this work |
