# Supplementary material for: Trans-Translation in Helicobacter pylori: Essentiality of Ribosome Rescue and Requirement of Protein Tagging for Stress Resistance and Competence
Source: PLoS One. 2008 Nov 26;3(11):e3810. doi: 10.1371/journal.pone.0003810 (PMC2584231; doi:10.1371/journal.pone.0003810)
Supplement: Table S2 — (0.19 MB DOC) [file pone.0003810.s002.doc]

Supplementary material

Table S2: Oligonucleotides used in this study

| **Target region** | Name | **Restriction site** | **Sequence (5'-3')** |
| --- | --- | --- | --- |
| **Cloning into pILL2150** | | | |
| *ssrA* | H265 | *Bam*HI | 5'-CGC*GGATCC*CAACGCCACCTTCTAAAACGAGAG-3' |
|  | H266 | *Spe*I | 5'-GG*ACTAGT*GATTTTTAATGATTCATTGCATC-3' |
| *smpB* | H225 | *Spe*I | 5'-GG*ACTAGT*AGGAAGAGAATAATGAAACTCATTGCCAGCAAC-3' |
|  | H226 | *Bam*HI | 5'-CGC*GGATCC*TTATCCTTTAAAGTGGTGTTTTAAATCAGCAAG-3' |
| **Chromosomal inactivation of *smpB* by three step PCR (3T-PCR)** | | | |
| 500 bp upstream from *smpB* | H227 | - | 5'-CTCAAGCCGGATTAGGCGGTGGGAGCACTG-3' |
|  | H228 | - | 5'-GTTAGTCACCCGGGTAC TCATTCGCCCCCTTTTTGCATAGG-3' |
| 500 bp downstream from *smpB* | H229 | - | 5'-TACCTGGAGGGAATAATGAAAGAAATGGTAGATTATGGG-3' |
|  | H230 | - | 5'-GGCTTTGCTATAAATCTCATGGGATTGCGTG-3' |
| **Chromosomal inactivation of *hp1248* by three step PCR (3T-PCR)** | | | |
| 500 bp upstream from *hp1248* | H214 | - | 5'-GATCCCTAAACCATTCGCTCCTCTAGTAG-3' |
|  | H215 | - | 5'-GTTAGTCACCCGGGTACCTTCACCCTGTCAAAAAGGCTC-3' |
| 500 bp downstream from *hp1248* | H216 | - | 5'-TACCTGGAGGGAATA*ATG*GAGCATGCCTTAAAAATAGCC-3' |
|  | H217 | - | 5'-CCTTTCTGTGATTTCGCCTCTAACTTGCCC-3' |
| **Chromosomal inactivation of *ssrA* by suicide vector pILL796** | | | |
| 500 bp upstream from *ssrA* | H274 | *Cla*I | 5'-CC*ATCGAT*CGGGGTTTTAAAGGCTAAAGCCCCTAATTG-3' |
|  | H253 | *Kpn*I | 5'-GTTAGTCACCCG*GGTACC*ATTGGCGCACAATATCAATG-3' |
| 500 bp downstream from *ssrA* | H273 | *Bam*HI | 5'-GGGAATAATGACCCGG*GGATCC* TCTTATTAAAAAGTATCATTTTTAAGCG-3' |
|  | H275 | *Pst*I | 5'-AA*CTGCAG*GGCTTTTCTCAATTCGGCTTCTAGTTGGGC-3' |
| *aphA-3* (Kn^R^) | pUNI | - | 5´-TAATACGACTCACTATAGGG-3´ |
|  | pRev | - | 5´-ACCCATGACCTTATTACCAACCTC-3´ |
| **Site directed mutagenesis on *ssrA*** | | | |
| SsrA^smpB^ | H267 | - | 5'-GCGCTTGGCATGCTATCTGTGCGACAAGAAATCTTAAGAAATCCAAG-3' |
|  | H268 | - | 5'-CGCACAGATAGCATGCCAAGCGCTGCTTGTAAAACAGC-3' |
| SsrA^wobble^ | H259 | - | 5'-CGCTTAAAAATGATACTTTTTAATATGGTG-G*G*G-CTGTGGGG-3' |
|  | H264 | - | 5'-AAAAAGTATCATTTTTAAGCGTATTTAAGGAAGGTAAATG -3' |
| SsrA^resume^ | H257 | - | 5'-CTTTAGCGTAAGCTGGAGCGTAATCTGTGTTGTT*TTA*AGTTATTTTTG-3' |
|  | H258 | - | 5'-GATTACGCTCCAGCTTACGCTAAAGCTGCGTGAGTTAATCTC- 3' |
| SsrA^DD^ | H255 | - | 5'-CTAATCAGTCCAGCTCCAAAAGGAGATTAACTCAGTCATCTTTAGCGTAAG-3' |
|  | H256 | - | 5'-CCTTTTGGAGCTGGACTGATTAGAATTTCTAGCGTTTTAATC-3' |
| SsrA^STOP^ | H380 | - | 5’ – gcagctttagcgtaagctggagcgtaatctgtTCATtAtacagttatt – 3’ |
|  | H381 |  | 5’ – ctccagcttacgctaaagctgcgtgagttaatctccttt – 3’ |
| **Insertion of the SsrA^DD^ and SsrA^STOP^ mutations on the chromosome of *H. pylori* strains by three step PCR (3T-PCR)** | | | |
| 500 bp upstream from *ssrA* | H276 | *Cla*I | 5'-CC*ATCGAT*CGGGGTTTTAAAGGCTAAAGCCCCTAATTG-3' |
|  | H277 | - | 5'-GTTAGTCACCCGGGTAC CATTGGCGCACAATATCAATGTTTTCATCTTTAGGC-3' |
| Mutated *ssrA* | H278 | - | 5'-TACCTGGAGGGAATAATGTGATTTTTAATGATTCATTGCATCTTGTTAGCAAAA GTTAGC-3' |
|  | H265 | *Bam*HI | 5'-CGC*GGATCC*CAACGCCACCTTCTAAAACGAGAG-3' |
| **Cloning of *hypB-TAP* with or without stop codon into vector pILL2150** | | | |
|  | H359 | *spe*I | 5'-GG*ACTAGT*cgtttaattaagtgcgctacgatacg-3' |
| *hypB-TAP* with stop codon | H340K | *Kpn*I | 5'- CG*GGTACC*GGTTGACTTCCCCGCGGAATTCGCG-3' |
| *hypB- TAP* without stop codon | H341K | *Kpn*I | 5'- CG*GGGTACC*TCAGGTTGACTTCCCCGCGGAATTCGCG–3’ |
| **Cloning of the *amiF* terminator downstream from *hypB-TAP* with or without stop codon** | | | |
| pILL2332 | H382 | *Kpn*I | 5’ – ACCtgaGGTACCtccctaaccttgcatttttgcCagaacccg – 3’ |
| pILL2332/2333 | H383 | *Kpn*I | 5’ – gggGGTACCaaaaaccagaacccttaaaaacgggttctGgcaaaaatgc – 3’ |
| pILL2333 | H384 | *Kpn*I | 5’ – ACCGGTACCtccctaaccttgcatttttgcCagaacccg – 3’ |
| **Insertion of the *hypB- TAP* fusion with or without stop codon on the chromosome of *H. pylori* strains by three step PCR (3T-PCR)** | | | |
| *hypB-tap* with or without stop codon | H410 | *-* | 5’ – gactttaagttttgcgtggtagagggcg – 3’ |
|  | H411 | *-* | 5’ – CACTCAAAACGATGAAATAGGGC – 3’ |
| *hp0898* amplification | H412 | *-* | 5’ – TAGCCCTATTTCATCGTTTTGAGTGtgatcagcggcgctcaaatctatgctcc – 3’ |
|  | H413 | *-* | 5’ – tcaaacgcgcttgtaacgataatacgcc – 3’ |
| **Riboprobes** | | | |
| *RNA 5S* | 5S Fw | *-* | 5’ – AGAGAAGAGGAACTACCC – 3’ |
|  | H346 | *-* | 5’ – CTAATACGACTCACTATAGGGAGAATTTCCCTATCCCTGCACCGACC – 3’ |
| *SsrA* | H244 | *-* | 5'-GGGGCTGACTTGGATTTCGACAG-3' |
|  | H245 | *-* | 5'-CTAATACGACTCACTATAGGGAGAGCTGTGGGGAATCGAACCCCAGTCC-3' |
